# Hepatitis A susceptibility parallels high COVID-19 mortality

**DOI:** 10.3906/sag-2007-133

**Published:** 2021-02-26

**Authors:** Faik SARIALİOĞLU, Fatma Burcu BELEN, Kadir Mutlu HAYRAN

**Affiliations:** 1 Department of Pediatric Hematology and Oncology, Faculty of Medicine, Başkent University, Ankara Turkey; 2 Department of Preventive Oncology, Institute of Cancer, Hacettepe University, Ankara Turkey

**Keywords:** Hepatitis A, COVID-19, vaccine, mortality

## Abstract

**Background/aim:**

COVID-19 has become the biggest health problem of this century. It has been hypothesized that immunity against hepatitis A virus (HAV) may provide protection from COVID- 19.

**Materials and methods:**

As of 10June 2020, the infection had spread to 213 countries, with 7.3 million people infected and 413,733 dead. This data was combined with the World Health Organization susceptibility classification on the worldwide prevalence of HAV, and the relationship between HAV susceptibility and COVID-19 mortality were analyzed.

**Results:**

When the data from 213 countries were analyzed, it was found that there was a significant increasing trend in COVID-19 mortality rates by HAV susceptibility (P <0.001). Using a cut-off of 200/million population, the mortality risk associated with living in a more susceptible country (medium/high) was 27.8 times higher (95% CI for OR: 3.6–213.2)

**Conclusion:**

The results of this study showed that, despite confounding factors in different countries, hepatitis A susceptibility of the population may have been correlated with COVID-19 mortality. This observation needs to be confirmed by further studies.

## To the Editor,

The COVID-19 pandemic has become the biggest health problem of this century. Several factors have caused the impact of the disease to vary among countries. It had been previously hypothesized that immunity against hepatitis A virus (HAV) may provide protection from COVID-19 [1], and herein, it was aimed to support this hypothesis with data. 

**Figure 1:**
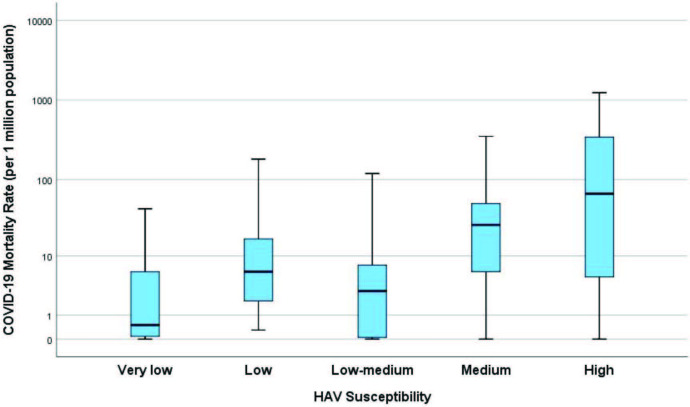
Box plots showing the increasing mortality trend by increasing HAV susceptibility in the 213 countries affected by the virus. If the data is limited to countries with more than 1 million population, 5 million population and 5000 tests/1 million population, the associated risk with increasing HAV susceptibility is even stronger (Supplementary file). The analyses do not take into account several confounding factors that may affect the death rates, including, but not limited to, socioeconomic status, accessibility of health services, and government pandemic policies. For example, Australia, New Zealand, Singapore, Japan, and South Korea are countries with excellent policies against the COVID-19 pandemic and have low mortality rates in the high susceptibility group. In contrast, countries with serious socioeconomic problems, such as Ecuador and Peru, have high mortality rates in the low susceptibility group. These are examples of a possible bias towards the null, while it is accepted that other sources of bias away from the null may exist.

**Figure 2:**
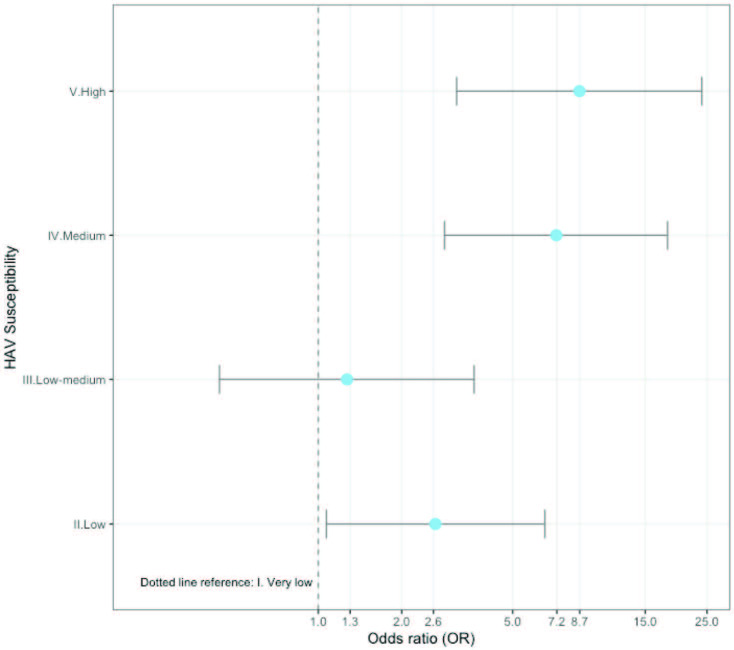
Increased risk of high mortality by increasing HAV susceptibility. High mortality is defined as having a mortality rate of more than 7/million (the median of 213 countries). The plotted ORs were from the univariate logistic regression model. The reference is the very low susceptibility group. Countries in the medium and high HAV-susceptible groups may be observed to have 7–8 times higher risk when compared to the reference (very low susceptibility) group.

As of 10 June 2020, the infection had spread to 213 countries, with 7.3 million people infected and 413,733 dead Worldometer (2020).Coronavirus COVID-19 Statistics [online]. Website https://www.worldometers.info/coronavirus/ [accessed 10 June 2020].. This publicly available data was combined with the World Health Organization susceptibility classification published in 2009 on the worldwide prevalence of hepatitis A [2]. Figures 1 and 2 show the significant increasing trend in COVID-19 mortality rates by HAV susceptibility (P <0.001, Jonckheere–Terpstra test). Moreover, using a cut-off of 200/million population,the mortality risk associated with living in a more susceptible country (medium/high) was 27.8 times higher (95%CI for OR: 3.6–213.2) (Table). 

**Table:** Risk associated with high or medium HAV susceptibility in the countries by subgroups.

		Mortality rate			Mortality rate			
		<200 per million	≥200 per million	OR (95% CI)	<7/million	≥7/million	OR (95%CI)	All countries
All countries(N = 213)	Very low, low, or low-medium	121	1	Reference	80	42	Reference	122
	Medium or high	74	17	27.8 (3.6–213.2)	26	65	4.76 (2.6–8.6)	91
	Total	195	18		106	107		213
Countries with >1 million population	Very low, low, or low-medium	106	1	Reference	70	37	Reference	107
	Medium or high	38	11	30.7 (3.8–245.7)	8	41	9.69 (4.1–22.8)	49
	Total	144	12		78	78		156
Countries with >5 million population	Very low, low, or low-medium	85	1	Reference	58	28	Reference	86
	Medium or high	26	10	32.6 (4.00–267.5)	6	30	10.4 (3.9–27.8)	36
	Total	111	11		64	58		122
Countries with >5000 tests/1 million population	Very low, low, or low-medium	50	1	Reference	26	25	Reference	51
	Medium or high	61	17	13.9 (1.8–108.4)	18	60	3.5 (1.6–7.4)	78
	Total	111	18		44	85		129

Association of high HAV susceptibility and high COVID-19 mortality holds in different subgroups. Two cut-offs were used: 7 (the median) and 200 (derived from the receiver operator curve analysis). OR: odds ratio showing risk in medium, high group when compared to the very low, low, low-medium group.

Several known facts supported the hypothesis. Although children constitute a very significant risk group for several respiratory viruses, they seemed to have been spared from COVID-19. The immunity of children against HAV (a virus with similar taxonomy to coronaviruses), acquired either by vaccination in developed countries or by infection in underdeveloped countries, may have contributed to this protection. The loss of immunity to HAV as the result of aging may have led to an increased COVID-19 morbidity in the elderly. 

The Diamond Princess ship experience also supported this theory. Of the 3711 passengers and crew members, 58% were over the age of 60. In total, 712 of the passengers were infected and 13 died (1.8% fatality).The asymptomatic infection rate was 57% in elderly individuals over the age of 60 [3]. Thus far, the reason for this extremely low fatality and unusually high asymptomatic infection rate has not been explained. The Centers for Disease Control and Prevention recommendation that all susceptible people traveling for any purpose, frequency, or duration to countries with high or intermediate HAV endemicity should be vaccinated before departure, which all notable cruise lines to Asia comply with, may have been one explanation [4].

We are aware that this ecological analysis is far from pointing to a causal relationship. However, given the simplicity and the very low risk/benefit ratio of the preventive measures, it is far from being negligible. 

We are proceeding with detailed analyses, to try and find confounders that may have led to this association, but with this letter, we are urging the medical community to help in challenging this hypothesis. If confirmed, the consequences of this simple discovery will be enormous.

## Informed consent

As this was an evaluation of publicly available open access data, informed consent was not required.
